# Rumen bacteria influence milk protein yield of yak grazing on the Qinghai-Tibet plateau

**DOI:** 10.5713/ab.20.0601

**Published:** 2020-12-01

**Authors:** Qingshan Fan, Metha Wanapat, Fujiang Hou

**Affiliations:** 1 State Key Laboratory of Grassland Agro-ecosystems, Key Laboratory of Grassland Livestock Industry Innovation, Ministry of Agriculture, College of Pastoral Agriculture Science and Technology, Lanzhou University, Lanzhou, 730000, China; 2 Tropical Feed Resources Research and Development Center (TROFREC), Department of Animal Science, Faculty of Agriculture, Khon Kaen University, Khon Kaen, 40002, Thailand

**Keywords:** Milk Protein Yield, Qinghai-Tibet Plateau, Rumen Microbiota, 16S RNA, Yak

## Abstract

**Objective:**

Ruminants are completely dependent on their microbiota for rumen fermentation, feed digestion, and consequently, their metabolism for productivity. This study aimed to evaluate the rumen bacteria of lactating yaks with different milk protein yields, using high-throughput sequencing technology, in order to understand the influence of these bacteria on milk production.

**Methods:**

Yaks with similar high milk protein yield (high milk yield and high milk protein content, HH; n = 12) and low milk protein yield (low milk yield and low milk protein content, LL; n = 12) were randomly selected from 57 mid-lactation yaks. Ruminal contents were collected using an oral stomach tube from the 24 yaks selected. High-throughput sequencing of bacterial 16S rRNA gene was used.

**Results:**

Ruminal ammonia N, total volatile fatty acids, acetate, propionate, and isobutyrate concentrations were found to be higher in HH than LL yaks. Community richness (Chao 1 index) and diversity indices (Shannon index) of rumen microbiota were higher in LL than HH yaks. Relative abundances of the Bacteroidetes and Tenericutes phyla in the rumen fluid were significantly increased in HH than LL yaks, but significantly decreased for Firmicutes. Relative abundances of the *Succiniclasticum*, *Butyrivibrio 2*, *Prevotella 1*, and *Prevotellaceae UCG-001* genera in the rumen fluid of HH yaks was significantly increased, but significantly decreased for *Christensenellaceae R-7* group and *Coprococcus 1*. Principal coordinates analysis on unweighted UniFrac distances revealed that the bacterial community structure of rumen differed between yaks with high and low milk protein yields. Furthermore, rumen microbiota were functionally enriched in relation to transporters, ABC transporters, ribosome, and urine metabolism, and also significantly altered in HH and LL yaks.

**Conclusion:**

We observed significant differences in the composition, diversity, fermentation product concentrations, and function of ruminal microorganisms between yaks with high and low milk protein yields, suggesting the potential influence of rumen microbiota on milk protein yield in yaks. A deeper understanding of this process may allow future modulation of the rumen microbiome for improved agricultural yield through bacterial community design.

## INTRODUCTION

Milk protein is considered an important milk component and a key economic trait. Many efforts have been made to increase milk protein yield [1], with advancements in either milk protein or milk yield via improvements in nutrition, management, and genetics [2]. Although the milking performance of cows is now at a relatively high level—average annual milk protein yield of cows in the United States exceeds 314 kg [2]—with the continuous increase in both global population and per capita demand for milk, further improvement in the production efficiency and environmental sustainability of cows has become an urgent task.

Ruminants can convert fibrous plant materials into edible meat and milk products for human consumption through rumen microbial fermentation [3,4]. The rumen microbiota mainly consists of bacteria, archaea, and eukarya. The bacteria comprise thousands of different species and approximately 60% of the microbial mass [5]. Microbial fermentation provides ~70% of the energy [6] and 60% to 85% [7] of the protein requirements of the dairy cow, indicating a critical need for maximizing rumen function and describing rumen microbiota. These fermentation end-products have a direct impact on the physiological parameters of animals, such as milk composition [8]. Experiments with groups of dairy cows fed the same diet showed substantial differences in production efficiency [1,9,10]. Although these differences are often ascribed to differences in animal genetics [11,12], evidence is accumulating that differences in efficiency are associated with differences in the composition of the ruminal microbial community [5,13,14].

The Qinghai-Tibet plateau (QTP), located in the southwest part of China, known as the Earth’s third pole, is the highest and largest plateau on the planet. The major role of land use on this plateau has been for grazing livestock since ancient times. It has been reported that more than 15 million yaks (*Bos grunniens*) are raised on the QTP of China, accounting for approximately 90% of the total yak population worldwide [15]. Yaks are one of the world’s most treasured domesticated livestock, known as an iconic symbol of Tibet and of high elevations because it can thrive well in extremely harsh environments [16]. Yaks provide essential products, such as milk, meat, hair, and cheese, to people living on the QTP [17]. For these reasons, yaks are an important livestock and represent the primary source of milk for 6.5 million Tibetans. Therefore, it is imperative to understand the rumen microbiota of yak cows and how they can influence milk protein yield. The main objective of this study was to compare the composition of rumen bacterial communities between yaks with high and low milk protein yield using high-throughput sequencing technology, and to assess whether the difference may lead to variable volatile fatty acid (VFA) production. We hypothesized that rumen microbiota differed in yaks with high and low milk protein yield, and that fluctuation in microbiota could affect their microbial fermentation metabolites, including VFAs which contribute to milk protein yield.

## MATERIALS AND METHODS

### Animal care

All trial procedures were strictly in accordance with regulations for the management of the experimental field approved by Lanzhou University (Nos. 2010-1 and 2010-2). All procedures for handling and caring for animals conform with China’s regulations on the protection and use of laboratory animals, and are approved by the Chinese Zoological Society.

### Study sites and yak management

The study commenced in August 2019 at Manrima village, Maqu County, Gannan Tibetan Autonomous Prefecture, Gansu Province, China (33°404′N, 101°5212′E; elevation 3,704 m), in the northeastern part of the QTP. During the experimental period, a mean annual temperature of 2.0°C and rainfall of 602 mm were recorded at the local agrometeorological information station. The soils were classified as alpine meadows. The vegetation is a typical alpine meadow and is dominated by *Saussurea hieracioides* and *Anaphalis lactea*. Associated species mainly include *Potentilla anserina*, *Halenia elliptica*, *Cerastium caespitosum*, *Anemone trullifolia*, *Anemone rivularis*, *Thalictrum alpinum*, *Swertia bimaculata*, and *Tibetia forrestii* [15,18].

A total of 57 healthy mid-lactation yaks were used. The yaks were managed under similar conditions (age, parity, time of lactation, and health status) and were not provided with any food supplements. The nutrient composition of the herbage is presented in Supplementary Table S1. The mating season is generally in August to September and females calve in April to May. Females can give birth to calves every year, but usually every two years. During the lactation period, the cow was separated from the calf overnight and milked once or twice daily, but not fully so that the calf could get some milk. When milking, the yak was separated from the calf and secured with a rope. Calves were allowed to suck to initiate milk letdown, and then the yaks were hand-milked. Milk yields were recorded for 7 consecutive days, and milk samples were collected and measured on the seventh day. Afterwards, 12 high protein yield (high milk yield and high milk protein content, HH) yaks and 12 low protein yield (low milk yield and low milk protein content, LL) yaks, with significantly different milk yield and milk protein contents, were randomly selected from 57 healthy mid-lactation yaks. The statistical power of 12 yaks within each group was regarded as milk yield and protein content being >2.81 kg/d and >5.72% for HH yaks and <1.58 kg/d and <4.76% for LL yaks, respectively (Supplementary Table S2). Samples from 20 randomly selected quadrats (50 cm×50 cm) from the vegetation on which the yaks grazed were collected and found to be mixed herbage. Inedible herbage samples were removed and edible herbage samples were oven dried to constant weight at 60°C for 24 h to determine dry matter (DM), ground using a mill, passed through a 1 mm sieve, and stored for further chemical analysis.

### Chemical composition measurements

For milk and herbage samples, the herbage DM was prepared by subjecting the samples for DM determination in an air-flow oven at 65°C for 72 h [19]. Nitrogen content was determined using the Kjeldahl method, and crude protein was calculated as N×6.38 for milk and N×6.25 for herbage. Ether extract (EE) was determined by the weight loss of DM after 8 h of extraction with ether in a Soxhlet extractor [19]. The lactose content of milk was determined using the Lane-Eynon method [20]. The fibrous fractions of neutral detergent fiber and acid detergent fiber contents were analyzed using methods outlined by Van Soest [21]. Milk components were measured in g/100 g milk and herbage in g/100 g DM.

### Sampling of rumen contents and measurement of fermentation variables

Ruminal contents (liquid and particulate herbage material) were collected using an oral stomach tube from the 24 yaks selected. This method has been used extensively in previous studies [16,22]. The first 50 mL of rumen fluid was discarded to avoid contamination from previous animas or its own saliva; this was followed by collection of 50 mL rumen fluid from each animal prior to grazing in the morning, and immediate pH measurement by pH meter (Model 144 PB-10, Sartorius Co., Goettingen, Germany). The rumen contents were filtered with four layers of woven gauze and divided into two portions for analysis of ruminal fermentation parameters and for DNA extraction. The VFA concentrations were determined with GC 3420 gas chromatograph fitted with HP-INNO as capillary column (30×0.32 mm). The concentration of NH_3_-N in the rumen was later analyzed using a specific visible spectrophotometry device (UV-VIS8500, Tianmei, Shanghai, China) [23].

### DNA extraction, sequencing, sequence processing, and analysis

Bacterial DNA was prepared and extracted from the digesta using an E.Z.N.A. Stool DNA kit (Omega Bio-TEK, Norcross, GA, USA). The quality and quantity of extracted DNA were measured using an ND2000 spectrophotometer (NanoDrop Technologies Inc., Wilmington, DE, USA). The V3–V4 region of the bacterial 16S ribosomal RNA gene was amplified using the universal primer set 338F (5-ACTCCTACGGGAGG CAGCAG-3) and 806R (5-GGACTACHVGGGTWTCTA AT-3) [24]. The barcode of the unique eight-base sequence of each sample was added to each primer for sample identification and determination. Polymerase chain reaction was conducted in triplicates as follows: an initial denaturing step at 94°C for 5 min, followed by 28 cycles at 94°C for 30 s, 55°C for 30 s, and 72°C for 60 s, and a final extension at 72°C for 7 min. Amplicons were extracted from 2% agarose gels, purified using the AxyPrep DNA Gel Extraction Kit (Axygen Biosciences, Union City, CA, USA) according to the manufacturer’s instructions, and quantified using the QuantiFluor-ST system (Promega, Madison, WI, USA). Purified amplicons were pooled in equimolar concentrations and paired-end sequenced (2×300 bp) on an Illumina MiSeq PE300 platform (Illumina, Inc., San Diego, CA, USA) according to the standard protocols. Sequences were sorted based on their unique barcode, followed by removal of barcode and primer sequences using QIIME (version 1.9.0). Raw tags were merged using FLASH (version 1.2.11) with default parameters [25]. Low-quality reads were eliminated using QIIME (version 1.9.0) [26]. Clean tags were compared to the Gold database using the UCHIME algorithm to eliminate chimera sequences; effective tags were obtained for further analysis. These effective tags were clustered into operational taxonomic units (OTUs) of ≥97% similarity using UPARSE (version 7.0) [27]. Representative sequences were classified into organisms using RDP classifier (version 2.2) based on the SILVA (SSU123) database. Alpha diversity analysis was performed by calculating the Chao1 index, Shannon index, phylogenetic diversity index (PD_whole_tree) and observed species index (observed_species) using QIIME (version 1.9.0). Principal coordinates analysis (PCoA) was used to compare treatments of samples based on the unweighted Uni-Frac distance metric [28]. The raw reads were deposited at NCBI (under BioProject accession ID: PRJNA656118, RUN: SRR 12437630-SRR12437653).

### Statistical analysis

The chemical composition of herbage and milk, ruminal fermentation parameters, relative abundance of bacteria, and the alpha diversity indices were analyzed using a completely randomized design by one-way analysis of variance (SAS Institute Inc, version 9.2, USA). Significant difference was declared at p<0.05. Microbial networks were used to statistically identify keystone taxa; the combined score of high mean degree, high closeness centrality, and low betweenness centrality was used as a threshold for defining keystone taxa in microbial communities [29]. The correlation heatmaps were generated using the R program heatmap package. The rumen microbiota functional pathways were predicted using Tax4Fun software based on 16S sequencing data [30].

## RESULTS

### Ruminal fermentation variables

Table 1 presents the rumen fermentation variables of yaks with different milk protein yields. The rumen NH_3_-N (p< 0.01), total VFA (p<0.01), acetate (p<0.01), propionate (p< 0.01), and isobutyrate (p<0.01) concentrations, as well as the proportion of isobutyrate (p<0.01) were higher in the rumen of HH yaks than in LL yaks (Table 1). We did not find a difference in ruminal butyrate and valerate between both groups.

### Ruminal bacterial composition

**Figure 1:**
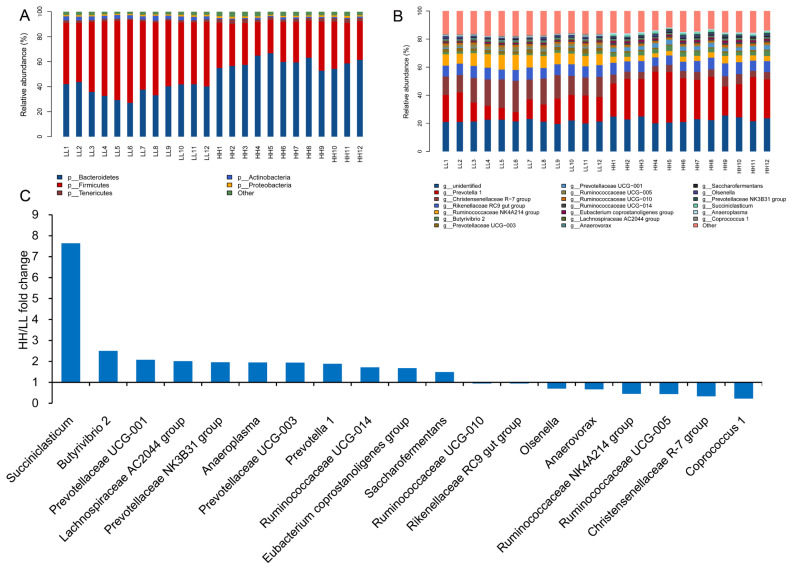
Comparison of the abundances of rumen bacterial phyla and genera between both groups. Comparison at the (A) phylum (relative abundance >1%) and (B) genus (relative abundance >0.1%) levels. (C) Fold change (HH/LL) in the relative abundances of bacterial genera (relative abundance >0.1% in at least 60% of the yaks within each group). HH, yaks with high milk yield and high protein content; LL, yaks with low milk yield and low protein content.

Overall, 1,650,786 V3–V4 16S rRNA sequence reads were obtained from the 24 samples in this study, with an average of 71,773 sequence reads per sample (minimum, 29,396; maximum, 143,850). Using these sequences, we identified 53,867 OTUs based on 97% nucleotide sequence identity between reads. As shown in Supplementary Figure S1A, 3,095 OTUs were shared between HH and LL yaks, which had 3,372 and 3,824 OTUs, respectively. Taxonomic analysis identified that the sequences belonged to 13 bacterial phyla and 174 bacterial genera, accounting for 99.64%±0.58% and 64.52% ±3.68% of the total bacterial sequences, respectively. The predominant bacterial phyla consisted of 5 taxa (with a relative abundance of >1%), Bacteroidetes (48.16%), Firmicutes (43.74%), Tenericutes (2.17%), Actinobacteria (2.01%), and Proteobacteria (1.14%) (Figure 1A). The predominant bacterial genera (with a relative abundance of >0.10%) consisted of 19 genera, with *Prevotella 1* (21.82%), *Christensenellaceae R-7* group (10.82%), *Rikenellaceae RC9 gut* group (7.84%), *Ruminococcaceae NK4A214* group (6.26%), and *Prevotellaceae UCG-001* (1.97%) (Figure 1B).

### Comparison of the rumen microbiota between yaks with different milk protein yields

**Figure 2:**
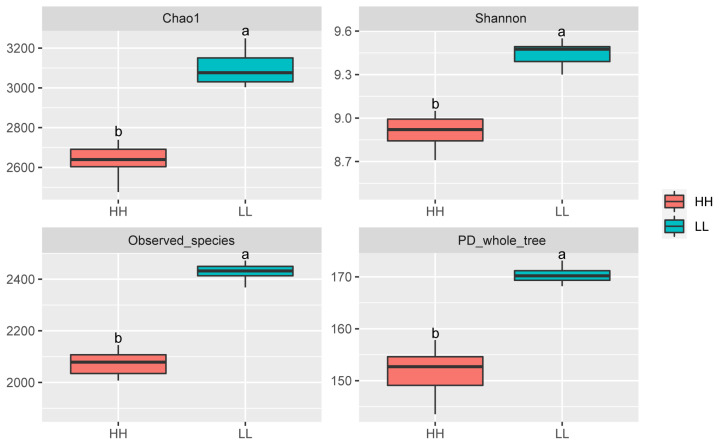
Alpha diversity indices of rumen bacteria in yaks between the two groups. Mean values with different superscripts are different at p<0.05 according to Duncan’s multiple-range test. HH, yaks with high milk yield and high protein content; LL, yaks with low milk yield and low protein content.

The alpha diversity index analysis is shown in Figure 2. The community diversity indices (Shannon index), community richness counts (Chao 1 estimator), observed_species, and PD_whole_tree of HH yaks were significantly decreased compared with those for LL yaks. PCoA plots based on unweighted UniFrac distance metrics revealed the differences in microbial diversity between HH and LL yaks (Supplementary Figure S1B). At the phylum level, the relative content of Bacteroidetes and Tenericutes were significantly increased in the rumen fluid of HH yaks compared with LL yaks. The relative content of Firmicutes was significantly decreased (Supplementary Table S3). At the genus level, the relative content of *Succiniclasticum*, *Butyrivibrio 2*, *Prevotella 1*, and *Prevotellaceae UCG-001* in the rumen fluid of HH yaks was significantly increased (Supplementary Table S3; Figure 1C). The relative abundances of *Christensenellaceae R-7* group and *Coprococcus 1* were significantly decreased.

**Figure 3:**
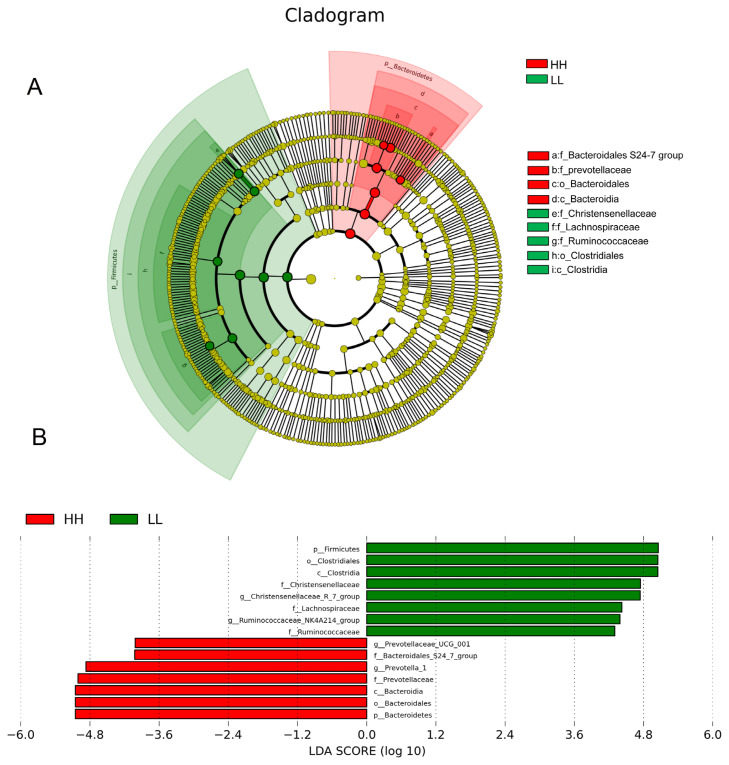
Microbial community differences between HH and LL yaks. (A) LEfSe cladogram comparing microbial communities between HH and LL yaks. Differences are represented by the color of the group where taxa are most abundant: red, taxa abundant in HH; green, taxa abundant in LL. (B) Histogram of LDA scores computed for each taxon ranging from phylum to genus. The LDA scores represent the difference in relative abundance with an exponential fold change of 10 between both communities, indicated by the significant difference in taxa. HH, yaks with high milk yield and high protein content; LL, yaks with low milk yield and low protein content; LDA, linear discriminant analysis.

We also performed LEfSe analysis to detect variations in the bacterial taxa composition. Figure 3A depicts a representative cladogram of the structure of the predominant microbiome, showing the most remarkable differences in taxa between HH and LL. The data indicated that four clades were more abundant in the HH group, including one class (Bacteroidia), one order (Bacteroidales), and two family (Bacteroidales S24-7 groups, prevotellaceae), while five clades were more abundant in the LL group, including one class (Clostridia), one order (Clostridiales), and three families (Christensenellaceae, Lachnospiraceae, Ruminococcaceae). The different bacterial taxa between HH and LL are shown in Figure 3B. When the microbial communities were compared between HH and LL, the most differentially abundant bacterial genera in HH were *Prevotella 1* and *Prevotellaceae UCG-001*, while *Christensenellaceae R-7* group and *Ruminococcaceae NK4A214* group were more abundant in LL. The genera *Prevotella 1* and *Christensenellaceae R-7* group were the most differentiated between communities, with an absolute LDA score factor of ~5.

**Figure 4:**
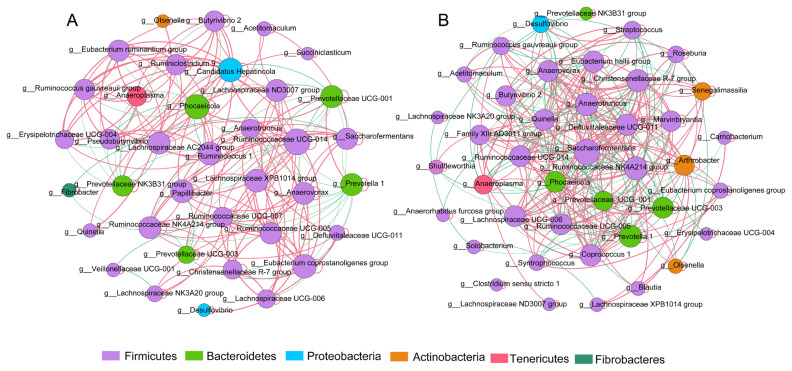
Interaction networks of rumen microbiota. A 16S rRNA gene-based correlation network of the rumen microbiota in (A) HH and (B) LL yaks, displaying statistically significant interactions with absolute correlation coefficients >0.6. Node size is scaled based on the overall abundance of each taxa in the microbiota. Red edge indicates positive correlation and green edge indicates negative correlation. HH, yaks with high milk yield and high protein content; LL, yaks with low milk yield and low protein content.

The microbial network was used to analyze the correlation between various genera and to statistically identify bacterial genera, which are keystone taxa regulating the fermentation process. The results showed that yaks with different milk protein yields had different correlations with rumen microflora (Figure 4A and 4B). The putative drivers of keystone taxa in the microbial communities of HH and LL yaks were defined with a combined score of high mean degree, high closeness centrality, and low betweenness centrality (Supplementary Table S4). The results showed that *Prevotellaceae UCG.003* in HH yak, and *Butyrivibrio 2*, and *Coprococcus 1* in LL yak can be considered as keystone taxa.

### Relationships of milk protein yield with rumen bacterial community

**Figure 5:**
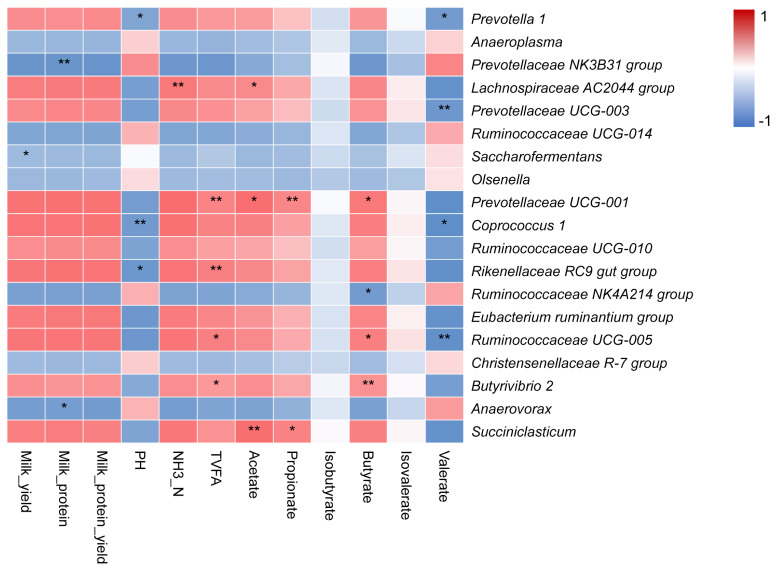
Spearman correlation between the milking traits, ruminal fermentation parameters, and main bacteria at genus level. TVFA, total volatile fatty acids; NH_3_-N, ammonia nitrogen. * and ** indicate different significant levels, 0.05 and 0.01, respectively.

We analyzed the correlation between the milking traits, ruminal fermentation parameters, and main bacteria at genus level through correlation analysis (Figure 5). The milk_yield was negatively correlated with the relative abundance of genera *Saccharofermentans*. The milk_protein content was negatively correlated with the relative abundances of genera *Prevotellaceae NK3B31* group and *Anaerovorax*. The NH_3_-N concentration was positively correlated with the relative abundance of genus *Lachnospiraceae AC2044* group. The TVFA concentration was positively associated with the relative abundances of the genera *Prevotellaceae UCG-001*, *Rikenellaceae RC9 gut* group, *Ruminococcaceae UCG-005*, and *Butyrivibrio 2*. The acetate molar proportion was positively correlated with the relative abundances of genera *Lachnospiraceae AC2044* group, *Prevotellaceae UCG-001*, and *Succiniclasticum*. The propionate molar proportion was positively correlated with the relative abundances of genera *Prevotellaceae UCG-001* and *Succiniclasticum*. The butyrate molar proportion was positively correlated with *Prevotellaceae UCG-001*, *Ruminococcaceae UCG-005*, together with *Butyrivibrio 2*, and was negatively associated with *Ruminococcaceae NK4A214* group abundance. The valerate molar proportion was negatively correlated with *Prevotella 1*, *Prevotellaceae UCG-003*, *Coprococcus 1*, and *Ruminococcaceae UCG-005* abundances.

### Tax4fun gene function prediction

**Figure 6:**
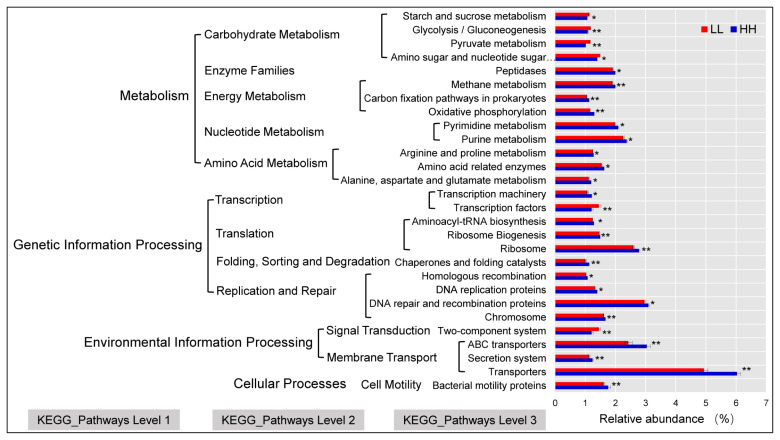
Functional predictions or rumen microbiota with significantly different KEGG pathways (* p<0.05, ** p<0.01) for HH and LL yaks. Levels 1, 2, and 3 KEGG pathways are represented. HH, yaks with high milk yield and high protein content; LL, yaks with low milk yield and low protein content; KEGG, Kyoto encyclopedia of genes and genomes.

Tax4Fun was used to predict the function of HH and LL yak rumen microbiota. The relative abundance of transporters (5.49%) was highest in HH and LL; DNA repair and recombination proteins (3.03%), ABC transporters (2.74%), ribosomes (2.69%), urine metabolism (2.32%), and pyrimidine metabolism (2.04%) were the second-most abundant. The Tax4Fun predictive software was used to enrich 54 predominant pathways (relative abundance >1%) at level 3 Kyoto encyclopedia of genes and genomes (KEGG) pathways. Among them, 28 pathways were significantly different in HH and LL yaks (p<0.05; Figure 6). Notably, the relative abundances of the transporters, DNA repair and recombination proteins, ABC transporters, ribosome, urine metabolism, pyrimidine metabolism, and amino and nucleotide sugar metabolism, significantly increased in HH yaks (p<0.05).

## DISCUSSION

Several factors influence milk protein yield: genetic factors account for about 25% [31] and management factors, including cow comfort, milking frequency, rationing system, and feeding management, account for the remaining 75% [32]. Here, we aimed to study differences in the rumen bacterial community composition of yaks with different milk protein yields. Rumen liquid was taken using an oral stomach tube, and it was demonstrated this method could replace the rumen cannulation method [16,33]. Thus, we collected rumen fluid from high and low milk protein yield yaks and investigated bacterial diversity via high-throughput sequencing.

In the current study, we identified varied bacterial diversity and specific rumen bacteria that may influence the milk protein yield of yaks. Comparison of the alpha diversity indices suggest a lower richness (Chao 1 index) and diversity indices (Shannon index) of rumen microbiota in yaks with high milk protein yield. The low richness of microbiota in the rumen has also been reported in cattle with higher feed efficiency [14,34], suggesting that HH yaks may have higher feed efficiency than LL yaks. Further, as feed-efficient animals are commonly considered to produce more VFAs [14], the higher VFA concentration in the rumen of HH yaks further supports that HH yaks may have higher feed-efficiency. However, it is noticeable that the ruminal VFA concentrations are the result of microbial production and host absorption. Future studies examining VFA absorption are needed to determine the relationship between microbial VFA production and host utilization, and their roles in high milk protein yield determination.

Bacteria are vital players in most of the feed degradation and fermentation processes [35], indicating that they play a more important role in determining host milk protein yield than other microbial taxa. The bacterial profiles of HH and LL yaks revealed differences in the relative abundances of rumen bacteria at various taxonomic levels, suggesting that specific bacteria might influence milk protein yield. For example, a 1.59-fold enrichment of the Bacteroidetes phylum was found in the rumen of HH yaks, with the genus *Prevotella 1* being the most abundant within this phylum (28.77% vs 14.86% in LL yaks). According to reports, the rumen genus, *Prevotella 1*, can degrade starch, monosaccharides, and other non-fibrous polysaccharides as energy substrates, and can produce succinate as the main fermentation end-product [36]. The abundance of *Prevotella 1* has been reported to be negatively associated with milk yield [5], but no correlation between *Prevotella 1* and milk yield and milk protein yield was found in the current study. Differences in research reports can be attributed to variations in host species, geographic location, diet composition, sampling time, and rumen bacterial diversity analysis methods. *Christensenellaceae R-7* group tended to be more abundant in LL than HH yaks. A previous study showed a negative relationship between *Christensenellaceae R-7* group and milk protein content [13], indicating that the abundance of this taxon negatively affects milk protein and milk protein yield. Bacteria from this genus have also been reported in human feces; these are strictly anaerobic, non-motile, non-spore-forming, gram-negative species, which produce acetic acid and small amounts of butyric acid as fermentation end-products [37]. However, no correlation between *Christensenellaceae R-7* group and VFA concentrations was found in the current study. As *Christensenellaceae R-7* group contains some species that may have varied functions, further investigations at deeper taxonomic levels are required to identify the linkages between *Christensenellaceae R-7* group species, VFA concentrations, and milk protein yield. In addition, the *Coprococcus 1* genus in the rumen has been reported to be positively correlated with the total feed efficiency of dairy cows [14] due to its ability to produce succinate, the precursor of propionate. Propionate is the main precursor for gluconeogenesis in the liver, which is vital for cow milk production. Based on higher rumen propionate concentration in HH than LL yaks, the relative abundance of *Coprococcus 1* was speculated to be higher in HH than LL yaks. However, higher abundances of *Coprococcus 1* were found in LL yaks, which was inconsistent with our speculation. This discrepancy may be because the taxonomic assessment was based only on the genus level.

Microbial communities play a role in ecosystem functioning [38], and correlations between microorganisms are complex in the rumen fermentation ecosystem. Possibly, certain species have a disproportionately larger impact on the community relative to their abundance [39]. This study first identified the keystone taxa with network topological properties in the rumen of yaks. The results showed that yaks with different milk protein yields have different correlation indices with rumen microflora, and the keystone genera identified were completely different between both groups of yaks, which further confirmed that rumen bacteria play a vital role in the regulation of milk protein yield.

The Spearman correlation analysis indicated that *Prevotellaceae UCG-001* might positively affect VFA concentrations (acetate, propionate, and butyrate). *Prevotellaceae UCG-001* is a genus composed of proteolytic, amylase hydrolysis, and hemicellulose hydrolysis bacteria. It is mainly distributed in the rumen of adult cows and can produce succinic acid and acetate [36]. The abundance of *Prevotellaceae UCG-001* has been reported to be positively associated with milk yield [40]. Although our results showed that *Prevotellaceae UCG-001* contributes to higher VFA concentrations, our study could not confirm whether members of this genus influence milk protein yield. Additionally, *Butyrivibrio 2* tended to be more abundant in HH than LL yaks. A previous study showed a positive relationship between *Butyrivibrio 2* and milk protein content [41], indicating that the abundance of this taxon positively affects milk protein and milk protein yield. *Butyrivibrio 2* is a fiber-digesting bacterium that also can digest starch to produce butyrate [42], indicating that the relative abundance of *Butyrivibrio 2* is closely related to butyrate concentration.

Surprisingly, the abundance of the genus *Succiniclasticum* was >7.6-fold higher in HH (1.07%) than LL (0.14%) yaks. Bacterial genera belonging to the Succinivibrionaceae family ferment carbohydrates to produce succinate (a precursor of propionate) and acetate, and their high abundance has been linked to lower methane emissions, accompanied by improved acetate and hydrogen production [43]. Recently, it was shown that Succinivibrionaceae was positively correlated with the total feed efficiency of dairy cows [14], and that it competed with methanogens for the hydrogen required to make succinate, a precursor for propionate [44]. *Succiniclasticum* plays an important role in slowing down methane and propionic acid production and providing energy for host tissue metabolism [45]. Propionate is the main precursor for gluconeogenesis in the liver, which is vital for cow milk production [46]. In our study, the higher propionate concentration in HH than LL yaks, and corresponding positive correlations between *Succiniclasticum* and propionate concentration indicated that the *Succiniclasticum-*enriched microbiome in HH yaks resulted in a fermentation shift toward the production of end products (propionate), contributing to high milk yield. However, this speculation needs to be further verified by the characterization of the microbial metabolism and intermediate metabolites in yaks with high milk protein yield.

Microorganisms have an impact on the body’s immunity, nutrient degradation and absorption, and enzyme metabolism [47]. In the current study, we used Tax4Fun to predict the function of the yak rumen microbial community. Spectacularly, in KEGG pathways level two, genes involved in carbohydrate metabolism were enriched in LL yaks, including “Pyruvate metabolism”, “starch and sucrose metabolism”, and the downstream pathway of “glycolysis” that converts glucose to pyruvate, indicating that more hydrolytic products and pyruvate might be generated by the LL microbiome due to a stronger ability to degrade carbohydrates. This result suggests a decreased feed energy requirement in the form of VFAs during microbial fermentation in the LL microbiome [48,49]. Genes involved in energy metabolism were enriched in HH yaks, indicating that their rumen microbiota help hosts maximize nutrient uptake and energy extraction from indigestible plant ingredients, such as cellulose, by producing high levels of VFAs that provide the host with extra energy. In this study, the relative abundance of transporters was highest at KEGG pathway level three. KEGG orthology groups related to transporters were reported as the largest known protein family [50]. In addition, Hamana [51] demonstrated that the transport function is a barrier for protecting ruminants from the invasion of toxic substances. In the current study, the relative abundance of transporters was high in HH yaks, which might be the reason for the high milk protein content in HH yaks [52]. Nevertheless, our results were only based on predicted metagenomics and may not represent the actual function of rumen bacteria. Further studies should be conducted to directly sequence the yak rumen metagenome to explore the roles of these genes in HH and LL yaks. Also, considering the potential for under sampling of rumen contents (1 sample/yak) as a limitation of this study, we recommend adequate sampling (different sampling sites and time) for future studies, so as to offer more representative rumen bacterial community profiles.

## CONCLUSION

Rumen microbiota diversity, function, and abundance of some bacterial taxa significantly differed between yaks with high and low protein yields. Bacterial richness, diversity indices, and relative abundances of *Coprococcus 1* and *Lachnospiraceae AC2044* group were lower, whereas the relative abundances of *Succiniclasticum*, *Butyrivibrio 2*, *Prevotella 1*, and *Prevotellaceae UCG-001*, along with VFA concentrations, were higher in the rumen of high protein yield than low protein yield yaks. Nevertheless, future studies should be conducted to modulate the rumen by inoculating the microbiome from the rumen of yaks with high or low milk protein yield. These findings are potentially applicable in future modulation of the rumen microbiome for improved agricultural yield via bacterial community design.

## Figures and Tables

**Table 1:** Comparison of rumen fermentation variables in HH and LL yaks

Item	Group^1)^		SEM	p-value

	HH	LL		
pH	7.42	7.53	0.013	<0.01
Ammonia N (mg/L)	104.96	65.62	4.116	<0.01
VFA concentration (mmol/L)				
Total VFA	62.29	54.28	0.893	<0.01
Acetate	46.52	39.86	0.769	<0.01
Propionate	8.95	7.69	0.177	<0.01
Butyrate	5.21	5.08	0.147	0.678
Isobutyrate	0.73	0.48	0.026	<0.01
Valerate	0.47	0.43	0.011	0.110
Isovalerate	0.42	0.74	0.033	<0.01
VFA molar proportion (%)				
Acetate	74.65	73.43	0.397	0.128
Propionate	14.37	14.16	0.191	0.600
Butyrate	8.37	9.35	0.282	0.083
Isobutyrate	1.17	0.89	0.032	<0.01
Valerate	0.76	0.80	0.020	0.304
Isovalerate	0.68	1.36	0.072	<0.01
Acetate:propionate	5.21	5.23	0.097	0.943

SEM, standard error of the mean; VFA, volatile fatty acids.

1) HH, yaks with high milk yield and high protein content; LL, yaks with low milk yield and low protein content.
