# Supplementary material for: Rumen bacteria influence milk protein yield of yak grazing on the Qinghai-Tibet plateau
Source: Anim Biosci. 2020 Dec 1;34(9):1466–78. doi: 10.5713/ab.20.0601 (PMC8495338; doi:10.5713/ab.20.0601)
Supplement: Supplementary file 1 [file ab-20-0601-suppl.pdf]

Supplementary information for:

Rumen bacteria influence milk protein yield of yak grazing on the Qinghai-Tibet Plateau

Qingshan Fan[1], Metha Wanapat[2], and Fujiang Hou[1,*]

[1] State Key Laboratory of Grassland Agro-ecosystems, Key Laboratory of Grassland Livestock Industry Innovation, Ministry of Agriculture, College of Pastoral Agriculture Science and Technology, Lanzhou University, Lanzhou, 730000, China

[2] Tropical Feed Resources Research and Development Center (TROFREC), Department of Animal Science, Faculty of Agriculture, Khon Kaen University, Khon Kaen, 40002, Thailand

[*] **Corresponding author: Fujiang Hou**

Tel: +86-931-8913047, Fax: +81-931-8910979, E-mail: cyhoufj@lzu.edu.cn

Table S1. Ingredients and chemical composition (based on dry matter) of the diet offered the yaks

Ingredient	Content %	Nutrient indices[1]	Content %
Cyperaceae	28.6	DM	38.36
Poaceae	19.4	CP	11.86
Ranunculaceae	15.3	EE	1.85
Compositae	16.8	OM	89.32
Fabaceae	3.4	NDF	54.13
Others	16.5	ADF	32.22

[1] Nutrient levels are measured values. DM, dry matter; CP, crude protein; EE, ether extract; OM, organic matter; NDF, neutral detergent fiber; ADF, acid detergent fiber.

Table S2. Milk yield and composition of yaks

Item	Group[1]		SEM	*P*-value
	HH	LL		
Yield, kg/d				
Milk	2.96	1.48	0.154	<0.01
Protein	0.17	0.06	0.011	<0.01
Composition, %				
Fat	5.75	5.70	0.024	0.285
Protein	5.84	4.69	0.119	<0.01
Lactose	5.59	5.54	0.022	0.401
Total solids	16.64	16.66	0.018	0.059
Ash	0.84	0.79	0.018	0.151

SEM, standard error of the mean.

[1] HH, yaks with high milk yield and high protein content; LL, yaks with low milk yield and low protein content.

Table S3. Comparison of rumen bacterial phyla and genera[1] between HH and LL yaks

Bacterial taxa	Relative abundance, [2] %		SEM	*P*-value
	HH	LL		
Phylum level				
Bacteroidetes	59.18	37.14	2.501	<0.01
Firmicutes	32.64	54.83	2.525	<0.01
Tenericutes	2.65	1.68	0.128	<0.01
Actinobacteria	1.14	2.88	0.205	0.163
Proteobacteria	1.02	1.26	0.052	0.438
Genus level				
Firmicutes				
Christensenellaceae R-7 group	5.45	16.19	1.219	0.013
Ruminococcaceae NK4A214 group	3.88	8.64	0.532	0.138
Ruminococcaceae UCG-014	1.29	0.75	0.068	0.651
Ruminococcaceae UCG-010	1.18	1.27	0.028	0.397
Eubacterium coprostanoligenes group	1.14	0.68	0.053	0.263
Succiniclasticum	1.07	0.14	0.106	<0.01
Butyrivibrio 2	2.40	0.96	0.170	0.012
Ruminococcaceae UCG-005	0.87	1.98	0.128	0.194
Saccharofermentans	0.91	0.61	0.041	0.352
Anaerovorax	0.62	0.93	0.041	0.183
Lachnospiraceae AC2044 group	0.58	0.28	0.048	0.235
Coprococcus 1	0.15	0.67	0.057	0.042

Actinobacteria				
Olsenella	0.62	0.89	0.060	0.622
Bacteroidetes				
Prevotella 1	28.77	14.86	1.742	0.018
Rikenellaceae RC9 gut group	7.67	8.01	0.143	0.248
Prevotellaceae UCG-003	1.51	2.91	0.179	0.413
Prevotellaceae UCG-001	2.66	1.28	0.220	0.024
Prevotellaceae NK3B31 group	1.08	0.56	0.094	0.536
Tenericutes				
Anaeroplasma	0.77	0.39	0.051	0.742

SEM, standard error of the mean.

[1] Bacterial phyla and genera with relative abundances > 0.1% were regarded as detected and used for comparison.

[2] HH, yaks with high milk yield and high protein content; LL, yaks with low milk yield and low protein content.

Table S4. Comparison of Degree, Closness centrality, and Betweenness centrality between HH and LL yaks

HH				LL			
Id	Degree	Closness centrality	Betweenness centrality	Id	Degree	Closness centrality	Betweenness centrality
Prevotella 1	15	0.540984	8.8528	*Christensenellaceae R.7 group*	25	0.639344	16.270756
Christensenellaceae R.7 group	11	0.428571	6.259113	*Prevotella 1*	25	0.639344	20.342076
Ruminococcaceae NK4A214 group	15	0.540984	35.47137	*Ruminococcaceae NK4A214 group*	27	0.672414	29.746946
Prevotellaceae UCG.001	15	0.6	21.47697	*Butyrivibrio 2*	21	0.557143	4.678828
Ruminococcaceae UCG.014	20	0.673469	89.29058	*Ruminococcaceae UCG.005*	22	0.557143	7.793046
Eubacterium coprostanoligenes group	17	0.578947	43.50984	*Anaerovorax*	22	0.609375	7.973822
Saccharofermentans	14	0.568966	33.20013	*Ruminococcaceae UCG.014*	22	0.573529	9.569304
Ruminococcaceae UCG.005	14	0.532258	34.66896	*Coprococcus 1*	23	0.573529	5.654305
Anaerovorax	15	0.540984	8.8528	*Saccharofermentans*	32	0.735849	74.58248
Lachnospiraceae XPB1014 group	16	0.559322	12.36309	*Family XIII AD3011 group*	19	0.541667	23.196338
Papillibacter	16	0.611111	26.61981	*Defluviitaleaceae UCG.011*	23	0.6	10.900387
Lachnospiraceae UCG.006	10	0.417722	0.611111	*Eubacterium hallii group*	23	0.609375	9.068266
Anaerotruncus	11	0.507692	11.7765	*Lachnospiraceae UCG.006*	22	0.6	8.100012
Ruminococcaceae UCG.007	12	0.5	8.372922	*Ruminococcus gauvreauii group*	14	0.5	40.095274
Prevotellaceae UCG.003	9	0.532258	3.420868	*Anaeroplasma*	17	0.534247	3.828954
Fibrobacter	4	0.392857	0	*Anaerotruncus*	26	0.661017	34.246495
Phocaeicola	18	0.578947	43.80598	*Quinella*	22	0.6	55.393745
Ruminococcus 1	16	0.559322	37.88039	*Phocaeicola*	22	0.609375	44.631367
Lachnospiraceae ND3007 group	15	0.6	61.70759	*Marvinbryantia*	21	0.6	13.808135
Candidatus Hepatincola	17	0.568966	33.04405	*Prevotellaceae UCG.003*	22	0.590909	12.622021
Prevotellaceae NK3B31 group	13	0.434211	0.650392	*Prevotellaceae UCG.001*	18	0.565217	19.527861
Butyrivibrio 2	13	0.5	6.542305	*Roseburia*	12	0.527027	40.007875
Anaeroplasma	12	0.417722	0.465909	*Senegalimassilia*	14	0.4875	1.008171
Erysipelotrichaceae UCG.004	10	0.4125	0.184483	*Streptococcus*	15	0.52	2.994827

Pseudobutyrivibrio	12	0.428571	0.184483	*Arthrobacter*	20	0.619048	152.088095
Eubacterium ruminantium group	14	0.532258	10.14865	*Lachnospiraceae NK3A20 group*	3	0.232143	0
Ruminococcus gauvreauii group	13	0.434211	0.650392	*Olsenella*	7	0.4875	0
Ruminiclostridium 9	13	0.434211	0.650392	*Carnobacterium*	4	0.29771	0
Succiniclasticum	3	0.666667	0	*Lachnospiraceae XPB1014 group*	5	0.410526	108
Lachnospiraceae AC2044 group	15	0.55	23.56759	*Solobacterium*	5	0.3	38
Lachnospiraceae NK3A20 group	7	0.44	6.797534	*Acetitomaculum*	9	0.46988	0.761905
Olsenella	3	0.666667	0	*Desulfovibrio*	13	0.493671	5.621068
Quinella	4	1	1	*Eubacterium coprostanoligenes group*	9	0.46988	0.125
Acetitomaculum	5	0.464789	0.973011	*Syntrophococcus*	10	0.47561	0.285714
Defluviitaleaceae UCG.011	6	0.392857	32	*Shuttleworthia*	6	0.419355	38.076923
Desulfovibrio	3	0.284483	0	*Blautia*	6	0.464286	1
Veillonellaceae UCG.001	4	0.354839	0	*Prevotellaceae NK3B31 group*	3	0.29771	0
				Lachnospiraceae ND3007 group	3	0.336207	0
				Clostridium sensu stricto 1	2	0	0
				Anaerorhabdus furcosa group	3	0.378641	0
				Erysipelotrichaceae UCG.004	3	0.348214	0

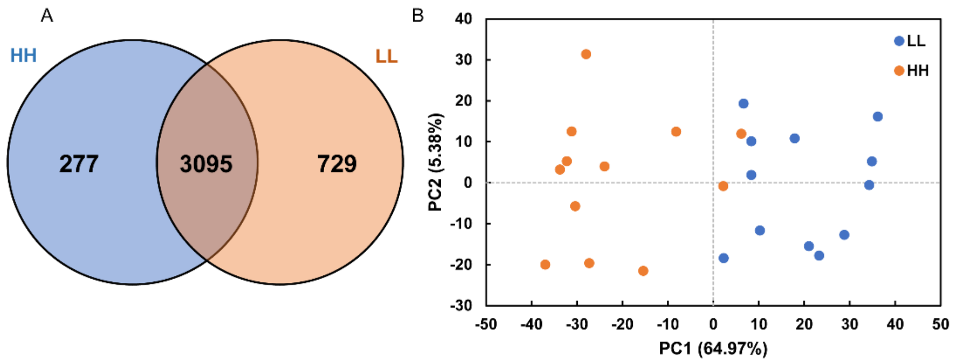

Figure S1. Differences in bacterial community diversity, richness, and OTUs between the two groups. (A) A Venn diagram showing the different and similar OTUs between both groups. (B) A principal coordinate analysis (PCoA) of the yak ruminal microbiota between both groups. HH, yaks with high milk yield and high protein content; LL, yaks with low milk yield and low protein content. OTU, operational taxonomic unit.
